# Electrolyte Imbalance Determination of a Vanadium Redox Flow Battery by Potential‐Step Analysis of the Initial Charging

**DOI:** 10.1002/cssc.201903485

**Published:** 2020-02-28

**Authors:** Kirstin Beyer, Jan grosse Austing, Barbara Satola, Timo Di Nardo, Marco Zobel, Carsten Agert

**Affiliations:** ^1^ Urban and Residential Technologies DLR Institute of Networked Energy Systems Carl-von-Ossietzky-Str. 15 26129 Oldenburg Germany; ^2^ VANEVO GmbH Johann-Hinrich-Engelbart-Weg 2 26131 Oldenburg Germany; ^3^ Innovation and Business Development EWE GASSPEICHER GmbH Moslestraße 7 26122 Oldenburg Germany

**Keywords:** batteries, capacity decay, imbalance detection, redox chemistry, vanadium

## Abstract

Vanadium redox flow batteries (VRFB) suffer from capacity fades owing to side reactions and crossover effects through the membrane. These processes lead to a deviation of the optimal initial average oxidation state (AOS=+3.5) of vanadium species in both half‐cell electrolytes. To rebalance the electrolyte solutions, it is first necessary to determine the current AOS. In this study, a new method was developed that enables an accurate determination of the AOS. A potential‐step analysis was performed with mixed electrolyte solutions of both half‐cells during the initial charging. The potential was recorded with a simple open‐circuit voltage (OCV) cell, and the potential‐steps were analyzed. A correlation between the duration of the potential plateaus in the OCV and the amount of vanadium ions of a certain oxidation state in the half‐cell electrolytes was found and used to precisely determine the AOS with a maximum error of 3.6 %.

## Graphical abstract

**One big step**! A new method to quantify electrolyte imbalance in vanadium redox flow batteries is proposed. The key principle is a correlation between the duration of the potential plateaus in the open‐circuit voltage during initial charging and the amount of vanadium ions of a certain oxidation state in the half‐cell electrolytes. By applying this potential‐step analysis, the average oxidation state can be determined with a maximum error of 3.6 %.

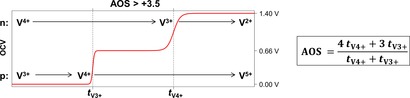

## Introduction

The increasing share of renewable energy production comes with the need to overcome the fluctuations in electricity production. In this context, energy‐storage devices such as batteries are considered to play an increasingly vital role. Amongst batteries, redox flow batteries have interesting properties such as a very high cycle life, the absence of flammability, and perfect suitability for large‐scale stationary storage applications owing to their setup.^1^, ^2^

The most mature redox flow battery is the vanadium redox flow battery (VRFB), which has been investigated since the 1980s.^3^ This redox flow battery uses a vanadium electrolyte with different oxidation states in both half‐cells. The redox couples are V^2+^/V^3+^ in the negative electrolyte and VO^2+^/VO_2_^+^ (hereinafter referred to as V^4+^/V^5+^) in the positive electrolyte. Nevertheless, there are mechanisms that lead to irreversible capacity fades of VRFBs, which are still under investigation.^4^, ^5^, ^6^ Despite the aging mechanisms that occur in the stacks (e.g., corrosion, increase of electrical resistance), the capacity of each electrolyte is crucial for the overall capacity and therefore the state of health of a VRFB. Side reactions such as hydrogen and carbon dioxide evolution, oxidation of species by oxygen from air,^7^, ^8^ crossover processes through the membrane, and volume changes during operation can lead to imbalanced capacities of the two electrolytes.^9^ However, the maximum capacity of the VRFB is limited by the electrolyte with the lowest capacity. Therefore, the optimal overall capacity is achieved if both electrolytes have the same capacity. For VRFBs, this is the case if both electrolytes have the same volume, the same concentration of vanadium species, and an average oxidation state (AOS) of the vanadium species of +3.5 (which means that the electrolyte consists of 50 % V^3+^ and 50 % V^4+^ species only).

There are straightforward methods that can be used to balance the volume and vanadium concentration of the electrolytes (mixing, splitting the electrolytes in two equal portions), whereas balancing the AOS is more demanding: for example, chemical treatment with reducing agents,^10^, ^11^ mixing with extra vanadium electrolytes,^12^ or electrochemical methods such as electrolysis^13^, ^14^ have been investigated. Determining the AOS is required before conducting compensation measures for imbalanced AOS. Different methods for analyzing the AOS in VRFBs have been presented in the literature. The oxidation state of the two electrolytes (or of a mixture of both electrolytes) can be determined by potentiometric titration.^15^ However, for this method, samples of the electrolytes must be extracted and analyzed ex situ. This procedure is time‐consuming and needs additional equipment, making it unsuitable for industrial applications. A more convenient procedure is the determination of the concentrations of the vanadium species by analyzing the UV/Vis absorption spectra of the electrolytes.^8^, ^16^, ^17^, ^18^ On‐line monitoring is possible by using flow‐through cuvettes, which provides additional information about the state of charge of the VRFB. Li et al. have already proposed an electrolyte recovery based on UV/Vis measurement and electrolysis.^19^ Despite the mentioned advantages, extra equipment is still needed. Furthermore, the calibration is quite tedious, especially in case of the positive electrolyte, in which the V^5+^ absorption does not follow Beer's Law.^17^ Another approach utilizes a special open‐circuit voltage (OCV) cell with three half‐cells.^20^ With this cell it is possible to determine the current oxidation state in each half‐cell from the measured OCV and the given AOS. Although this method is “noninvasive” and suitable for on‐line determination of the electrolytes, a special electrochemical cell with three half‐cells is needed. Additionally, it is only applicable for monitoring the current oxidation state, whereas the AOS has to be determined by potentiometric titration.

In this study, we developed a new method that enables the AOS of the electrolytes to be determined by using a standard OCV cell. The analysis of the potential steps during the initial charging of mixed electrolytes allows an accurate determination of the AOS. This procedure is straightforward and allows the AOS in VRFB to be determined without the need for special apparatus because most VRFB‐systems are already equipped with an OCV cell for monitoring the state of charge. The suitability of the method is investigated in a small lab‐scale cell and verified under real operation conditions in a 4‐cell short‐stack VRFB‐system.

## Results and Discussion

### Potential‐step analysis during initial charging of mixed electrolytes

Knowing that every combination of redox couples in the vanadium electrolyte has its characteristic potential difference, a potential‐step analysis during the initial charging of mixed electrolytes can be utilized to determine the AOS. An overview of half‐cell potentials and potential differences in a VRFB is shown in Table 1.

**Table 1:** Overview of standard potentials *E*_0_ at different oxidation states in negative and positive electrolyte and their resulting potential difference Δ*E*.^21^

Negative electrolyte [V]	Positive electrolyte [V]	Potential difference (Δ*E*) [V]
*E* ^0^ ${}_{V{}^{3+},V{}^{4+}}$ =0.337	*E* ^0^ ${}_{V{}^{3+},V{}^{4+}}$ =0.337	0.000
*E* ^0^ ${}_{V{}^{2+},V{}^{3+}}$ =−0.255	*E* ^0^ ${}_{V{}^{3+},V{}^{4+}}$ =0.337	0.592
*E* ^0^ ${}_{V{}^{3+},V{}^{4+}}$ =0.337	*E* ^0^ ${}_{V{}^{4+},V{}^{5+}}$ =1.000	0.663
*E* ^0^ ${}_{V{}^{2+},V{}^{3+}}$ =−0.255	*E* ^0^ ${}_{V{}^{4+},V{}^{5+}}$ =1.000	1.255

Starting with identical electrolytes with equal volumes and AOS in both half‐cells, a potential difference of approximately 0 V is expected. During the charging process, alteration of the oxidation states in both half‐cells occurs. In the negative half‐cell electrolyte, the vanadium ions are reduced from V^4+^ to V^3+^ in a first step, and subsequently, after conversion of all V^4+^ to V^3+^, V^3+^ is reduced to V^2+^ in a second step. In the positive half‐cell electrolyte, the vanadium ions are oxidized from V^3+^ to V^4+^ in a first step, and subsequently from V^4+^ to V^5+^ in a second step. Depending on the AOS of the initial electrolyte, the second reduction/oxidation step (V^3+^→V^2+^ or V^4+^→V^5+^, respectively) will begin at the same time as the first step (for AOS=+3.5) or at a different time (for AOS≠+3.5).

**Figure 1:**
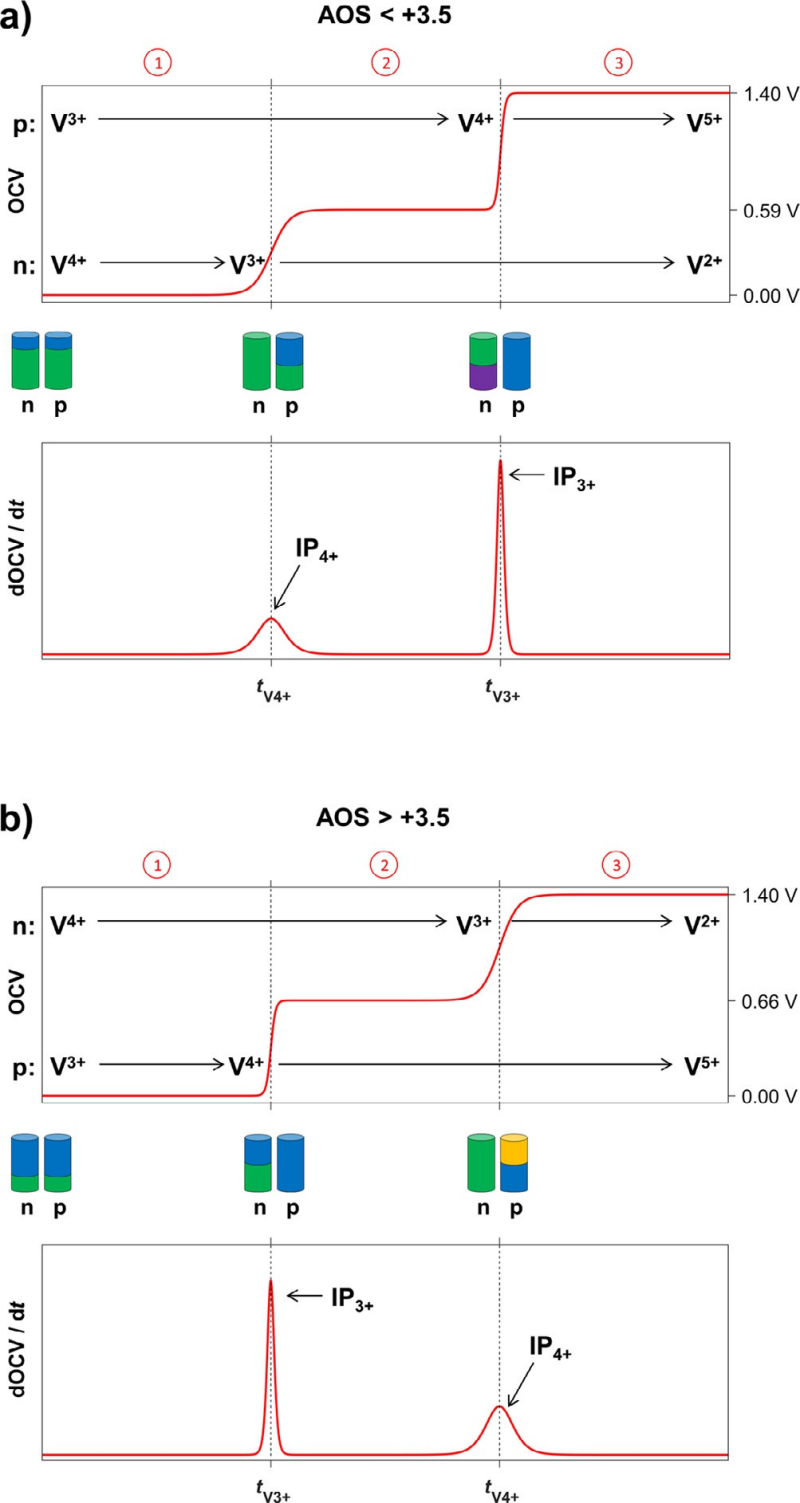
Schematic representation of the potential steps during the initial charging of mixed imbalanced electrolytes with (a) AOS<+3.5 and (b) AOS>+3.5, in which three plateaus of different present redox couples can be defined. (n): negative half‐cell electrolyte; (p): positive half‐cell electrolyte; IP_3+_: inflection point of total V^3+^→V^4+^ oxidation within the positive half‐cell electrolyte; IP_4+_ inflection point of total V^4+^→V^3+^ reduction within the negative half‐cell electrolyte. *t* ${}_{V{}^{4+}}$ and *t* ${}_{V{}^{3+}}$ are defined as the times of the inflection points IP${}_{V{}^{4+}}$ and IP${}_{V{}^{3+}}$ , respectively.

For an AOS=+3.5 the oxidation states of both half‐cell electrolytes will simultaneously change in such a way that no intermittent potential plateau can be observed, and the measured OCV will increase directly from approximately 0 to 1.4 V (1.255 V is the theoretical potential difference; the formal potential is 1.4 V^11^). However, for imbalanced electrolytes, the second vanadium reduction/oxidation step is reached earlier in one half‐cell than in the other one. Therefore, either a potential plateau of 0.592 V (for AOS<+3.5) or 0.663 V (for AOS>+3.5) is expected owing to the cell potentials caused by the present redox couples (Table 1). When the second oxidation/reduction step is also reached in the other half‐cell, the potential difference between both half‐cells increases to approximately 1.4 V. A schematic diagram of the development of the OCV over time during the initial charging of mixed imbalanced electrolytes in a VRFB is shown in Figure 1 for an AOS<+3.5 (a) and AOS>+3.5 (b), as well as their derivations. Three plateaus occur in both cases. Each plateau is separated by one of the above‐mentioned potential steps. The first and third plateau have the same potential for every AOS, approximately 0 V in the first part and approximately 1.4 V in the third part. Therefore, the second plateau is characteristic for the determination of the AOS.

An AOS<+3.5 describes an overall excess of V^3+^ ions compared with V^4+^ ions in the mixed electrolyte. During the first plateau the reactions from V^4+^ to V^3+^ (negative electrolyte) and from V^3+^ to V^4+^ (positive electrolyte) occur simultaneously. At *t* ${}_{V{}^{4+}}$ all V^4+^ ions in the negative electrolyte are converted to V^3+^, and the predominant reaction in the negative electrolyte changes to further reduction of V^3+^ to V^2+^. Until *t* ${}_{V{}^{3+}}$ is reached, there are still V^3+^ ions present in the positive electrolyte. When *t* ${}_{V{}^{3+}}$ is reached, all V^3+^ ions are converted to V^4+^ ions in the positive electrolyte, and the oxidation of V^4+^ to V^5+^ begins. Hence, the times *t* ${}_{V{}^{4+}}$ and *t* ${}_{V{}^{3+}}$ characterize the ratio of V^3+^ and V^4+^ ions in the electrolytes. *t* ${}_{V{}^{4+}}$ is proportional to the amount of V^4+^ ions in the negative half‐cell, whereas *t* ${}_{V{}^{3+}}$ correlates with the amount of V^3+^ ions in the positive half‐cell. Supposing equal electrolyte volumes and equal overall vanadium concentrations in both half‐cells at the beginning of the initial charging, the AOS can be calculated with Equation (1). *t* ${}_{V{}^{4+}}$ and *t* ${}_{V{}^{3+}}$ are defined as the times of the inflection points IP${}_{V{}^{4+}}$ and IP${}_{V{}^{3+}}$ (Figure 1), respectively.

(1) $$\mathrm{AOS}=\frac{4\;t{}_{V{}^{4+}}+3\;t{}_{V{}^{3+}}}{t{}_{V{}^{4+}}+t{}_{V{}^{3+}}}$$

In the case of AOS>+3.5, an overall excess of V^4+^ is present in the mixed electrolyte. Analogously, the first potential step is caused by an oxidation of all V^3+^ to V^4+^ in the positive half‐cell electrolyte and the beginning of further oxidation from V^4+^ to V^5+^. The second potential step results from the total reduction of all V^4+^ to V^3+^ in the negative half‐cell electrolyte followed by the beginning of the reduction of V^3+^ to V^2+^. Therefore, the reasons for the potential steps are opposite to those described for AOS<+3.5. Hence, *t* ${}_{V{}^{3+}}$ occurs prior to *t* ${}_{V{}^{4+}}$ . If the AOS equals exactly +3.5, both inflection points coincide. Only plateaus one and three would be present without any additional potential step. This means *t* ${}_{V{}^{4+}}$ equals *t* ${}_{V{}^{3+}}$ , and Equation (1) will result in AOS=+3.5.

To determine the inflection points, the temporal derivative of the OCV is calculated numerically, as illustrated in Figure 1 (lower part). The maxima in the derivative correspond to the inflection points in the OCV curve marking *t* ${}_{V{}^{4+}}$ and *t* ${}_{V{}^{3+}}$ . It was observed during various experiments that IP${}_{V{}^{3+}}$ is steeper than IP${}_{V{}^{4+}}$ for both cases. Therefore, the steeper inflection point is induced by the disappearance of V^3+^ ions in the positive half‐cell electrolyte. Likewise, the inflection point with the shallower slope results from the disappearance of V^4+^ ions in the negative half‐cell electrolyte. These observations might be caused by different vanadium‐ion transfer rates through the ion‐exchange membrane resulting from the combination of diffusion, migration, and/or electro‐osmotic convection processes. However, the analysis of the vanadium‐ion transfer rates or other possible effects are not within the scope of this paper and could be the subject of future studies. Nevertheless, the slope of the derivative supports the potential‐step analysis because it gives additional evidence for the presence of AOS<+3.5 or >+3.5. This is essential because the deviation over time in the voltage level of the second plateau might be too high to precisely match the expected voltage of either 0.592 V (for AOS<+3.5) or 0.663 V (for AOS>+3.5). Therefore, the average voltage level of plateau 2 cannot serve as information whether the AOS of the electrolyte is <+3.5 or >+3.5. However, by analyzing the derivative of the measured OCV, the AOS orientation (<+3.5 or >+3.5) can be determined from the slope of the inflection points. Nevertheless, the distinction between AOS<+3.5 and >+3.5 is not necessarily required in real‐life applications because there is only an occurrence of AOS with values ≥+3.5 under real operation conditions.^22^

### Validation experiments in lab‐scale single cell

**Figure 2:**
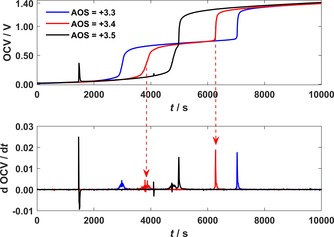
OCV and its temporal derivative during the initial charging of electrolyte samples of different AOS≤+3.5 in the lab‐scale single cell.

**Figure 3:**
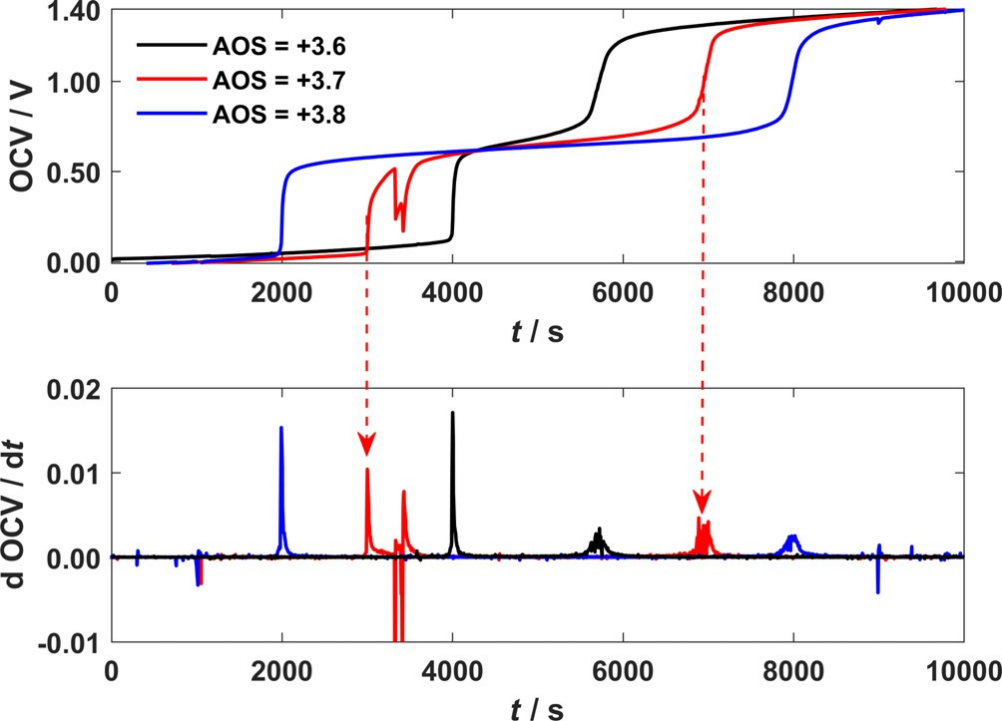
OCV and its temporal derivative during the initial charging of electrolyte samples of different AOS>+3.5 in the lab‐scale single cell.

Validation experiments of this new method were performed in a lab‐scale single‐cell VRFB. For several electrolyte samples with a predefined AOS, the OCV potentials were measured during initial charging, and the potential‐step analysis was applied. The obtained OCV potentials for AOS≤+3.5 and AOS>+3.5 are shown in Figures 2 and 3, respectively. As discussed above, three plateaus were observed in the OCV potential measurements for every sample, which were separated by the two inflection points IP${}_{V{}^{4+}}$ and IP${}_{V{}^{3+}}$ . The times *t* ${}_{V{}^{4+}}$ and *t* ${}_{V{}^{3+}}$ were determined from the corresponding peaks in the derivative of the OCV. As the electrolyte imbalance of the sample increased, the length of plateau 2 became longer and that of plateau 3 became shorter. Therefore, the length of plateau 3 corresponds to the capacity of the VRFB.^20^

For every sample, plateau 2 in the OCV potential was in the range between approximately 0.6–0.7 V. Because the deviation over time for this measured voltage level is too high to precisely match the expected voltage of either 0.592 V (for AOS<+3.5) or 0.663 V (for AOS>+3.5), the AOS value was determined by the slope of the inflection points. The steeper inflection point corresponds to a change of the vanadium redox pairs in the positive half‐cell electrolyte, whereas the shallower inflection point corresponds to a change of the vanadium redox pairs in the negative half‐cell electrolyte. Therefore, if the first inflection point in the derivative is flat (IP_4+_) and the second one is steep (IP_3+_), then the AOS is <+3.5 and vice versa. The appearance of additional peaks in the derivative is related to noise effects and can be identified by a correlating negative peak.

The obtained times *t* ${}_{V{}^{4+}}$ and *t* ${}_{V{}^{3+}}$ and the AOS results for the potential‐step analysis for every prepared sample are shown in Table 2. A good alignment between the prepared AOS and the measured AOS was observed. The maximum deviation was obtained for AOS=+3.4 with a deviation of 0.018. Compared with other methods, this is below the results of the UV/Vis‐based AOS detector proposed by Li et al., who found a maximum deviation of 0.039 by using their method.^13^

**Table 2:** Obtained times t_V4+_ and t_V3+_ and AOS results from the potential‐step analysis in a lab‐scale single cell for electrolyte samples with predefined AOS.

AOS prepared	*t* ${}_{V{}^{4+}}$ [s]	*t* ${}_{V{}^{3+}}$ [s]	AOS potential‐step	Δ AOS
+3.3	2980	7043	+3.297	0.003
+3.4	3879	6280	+3.382	0.018
+3.5	4738	4985	+3.487	0.013
+3.6	5725	4007	+3.588	0.012
+3.7	6890	3000	+3.697	0.003
+3.8	8015	2003	+3.800	0

Because the possible imbalance in the VRFB only spans a range of 0.5 (in the positive and negative direction in relation to +3.5), an error of 0.1 in the measured AOS means an error of 20 % in the imbalance detection. Accordingly, this new method can describe electrolyte imbalances with an error of 3.6 % compared with 5 % for potentiometric titration^22^ and 7.8 % for the UV/Vis detector^13^ (in 13 the authors mention an error of 1.28 %, which is the relative error; however, using the error calculation described above results in an imbalance accuracy of 7.8 %). This makes the method very reliable for the determination of AOS and further rebalancing steps.

### Application in a short‐stack VRFB‐system

**Figure 4:**
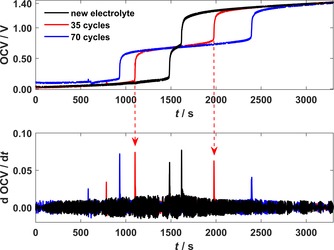
OCV and its temporal derivative during initial charging of the new and mixed electrolyte in a short‐stack VRFB‐system after different cycling stages.

To verify the method under real operation conditions, the potential‐step analysis of the initial charging for the determination of the AOS was applied to a four‐cell short‐stack VRFB‐system. After each 35 cycles, the electrolytes were mixed and reused. During the subsequent initial charging of the mixed electrolytes the AOS was determined by the potential‐step analysis and compared with the values determined by the UV/Vis method.^18^ The time‐dependent OCV during the initial charging and its temporal derivative for the new electrolyte after 35 and 70 cycles are shown in Figure 4.

For every measurement, a potential plateau at approximately 0.6–0.7 V was observed. The temporal length of the potential plateau increased with longer cycling of the electrolyte. This indicated an increase in the capacity imbalance between the positive and negative electrolyte. The short potential plateau for the new electrolyte indicated that its AOS was not equal to exactly +3.5; IP${}_{V{}^{4+}}$ occurred before IP${}_{V{}^{3+}}$ , which indicated an AOS<+3.5. This observation was in good agreement with the value of AOS=+3.49 derived from the data of the analysis certificate of the commercial electrolyte and the UV/Vis measurement of AOS=+3.497. After 35 and 70 cycles, the peak values in the derivatives were reversed, that is, IP${}_{V{}^{4+}}$ occurred after IP${}_{V{}^{3+}}$ . Hence, the AOS increased with the number of cycles towards values greater than +3.5. This increase of the AOS during cycling was expected and can be explained owing to side reactions that cause an irreversible oxidation of the overall electrolyte and thus represent a common problem in VRFBs.^23^

The obtained times *t* ${}_{V{}^{4+}}$ and *t* ${}_{V{}^{3+}}$ are shown in Table 3, together with the calculated AOS values and the values from the UV/Vis measurements. All AOS values from the potential‐step analysis are in the range of the AOS measured by UV/Vis analysis. The maximum deviation between two values was 0.029 for the AOS after 70 cycles. In terms of accuracy of the imbalance determination, this means an uncertainty of 5.8 % between the two different methods. Even though the data obtained from the short‐stack cycling is noisier than the data of the lab‐scale cell cycling, the evaluation is still accurate, and the values *t* ${}_{V{}^{4+}}$ and *t* ${}_{V{}^{3+}}$ were easy to identify. These findings emphasize the successful application of the potential‐step analysis even for larger systems, showing its high potential for automated maintenance algorithms. There is no need for any supplementary equipment other than the OCV cell and the voltage measurement, which comes on‐board most VRFB systems for state‐of‐charge determination.

**Table 3:** Obtained times *t*_V4+_ and *t*_V3+_ and AOS results from the potential‐step analysis of a short‐stack VRFB‐system compared with AOS values determined with the UV/Vis method.

Electrolyte sample	*t* ${}_{V{}^{4+}}$ [s]	*t* ${}_{V{}^{3+}}$ [s]	AOS potential‐step	AOS UV/Vis	Δ AOS
new electrolyte	1485	1681	+3.469	+3.497	0.028
mixed after 35 cycles	1997	1102	+3.644	+3.624	0.020
mixed after 70 cycles	2396	932	+3.720	+3.691	0.029

One constraint is that this new method is only applicable for fully mixed and equally parted electrolytes with an equal overall vanadium concentration. However, because ion exchange and electrolyte transfer between the two half‐cells take place during operation, an electrolyte mixing is recommended at periodic intervals to retract reversible capacity fading.^22^ This mixing can be directly utilized to apply the potential‐step analysis during the subsequent initial charging of the electrolytes.

## Conclusions

A new potential‐step analysis during initial charging of mixed electrolytes was developed for determining the average oxidation state (AOS) in vanadium redox flow batteries (VRFBs). The method consists of a straightforward process for determining the AOS from the measured open‐circuit voltage (OCV) curve. A correlation between the duration of the potential plateaus in the OCV and the amount of vanadium ions of a certain oxidation state in the half‐cell electrolytes was found and used to calculate the AOS. The potential‐step analysis was performed in situ with no need for any additional equipment except for an OCV cell, which is part of many VRFB systems for determining the state of charge. Moreover, this new method is simple, cost efficient, timesaving, and can predict the AOS with a high accuracy of 3.6 %. Precise AOS detection is important for the maintenance of a VRFB because knowledge about the electrolyte imbalance is essential for rebalancing and cycling strategies. Therefore, the proposed method has a high potential for automated maintenance algorithms in large‐scale VRFB systems.

## Experimental Section

### Setup lab‐scale cell experiments

The setup for the lab‐scale experiments consisted of an in‐house‐developed single‐cell assembly with an active area of 4×4 cm, two pumps (Simdos 10, KNF Flodos AG, Switzerland), two electrolyte tanks, and a standard two‐compartment OCV cell. Each half‐cell was connected by tubes in the following order: electrolyte tank, pump, the corresponding half‐cell of the OCV cell, and then the cell assembly. For the electrochemical measurements, a potentiostat/galvanostat (Solartron Analytical Modulab Pstat, Model 2100 A with Booster 12 V/20A) was utilized. A fumasep FAP‐450 (fumatech GmbH, Germany) membrane was used as an ion‐conductive separator for the half‐cells. Each half‐cell consisted of a SIGRACELL GFD 4.6 EA graphite felt (SGL Carbon SE, Germany), a SIGRACELL bipolar plate PV15 (SGL Carbon SE, Germany), and a copper plate as current collector. The felts were pretreated at 400 °C for 18 h in air atmosphere in an oven (P330, Nabertherm GmbH, Germany) and compressed by 20 % in the assembly. The electrolyte tanks were flushed with nitrogen during each experiment. To achieve a stable behavior of the membrane, the cell was first cycled 20 times by using a commercial vanadium electrolyte [OXKEM, 1.62 m vanadium sulfate (51 % V^3+^, 49 % V^4+^) in 2 m sulfuric acid] with a pumping rate of 100 mL min^−1^ in the range between 0.7–1.8 V (cell voltage) at ±93.75 mA cm^−2^. For the standard two‐compartment OCV cell, SIGRACELL bipolar plates PV15 (SGL Carbon SE, Germany) were applied as current collectors and a Nafion 117 (Ion Power GmbH, Germany) membrane was used as a separator with an active area of 2.36 cm^2^.

### Preparation of electrolyte solutions with defined AOS

Electrolyte samples with different AOS were prepared. Two electrolyte solutions (with AOS=+3 and AOS=+4) were obtained by charging the commercial electrolyte [OXKEM, 1.62 m vanadium sulfate (51 % V^3+^, 49 % V^4+^) in 2 m sulfuric acid, AOS=+3.49]. For this purpose, first a successive charging of 1050 mL electrolyte in each half‐cell was performed with 125, 93.75, 62.5, 31.25, and 6.25 mA cm^−2^ up to a cell voltage of 1.8 V to obtain fully charged electrolytes with oxidation states of V^2+^ and V^5+^. For the preparation of 1 L of 100 % V^3+^ electrolyte, 50 mL of the negative half‐cell electrolyte (V^2+^) was removed, and the system was discharged successively with −125, −93.75, −62.5, −31.25, −6.25, and −5 mA cm^−2^ until a cut‐off voltage of 0.8 V. The same procedure was repeated to prepare 1 L of 100 % V^4+^ electrolyte by removal of 50 mL in the positive half‐cell. From these two electrolyte solutions with known oxidation states of AOS=+3 and AOS=+4, six electrolyte samples with defined AOS of +3.3, +3.4, +3.5, +3.6, +3.7, and +3.8 were prepared by mixing the V^3+^ and V^4+^ electrolytes in the necessary ratio.

### Measurement of the AOS by potential‐step analysis in a lab‐scale cell

For the measurement of the AOS of the six electrolyte solutions, 80 mL of the defined AOS samples was filled into each electrolyte tank of the setup. The initial charging was performed with 80 mA cm^−2^ up to an OCV of 1.4 V. The OCV was recorded and evaluated by applying the potential‐step analysis. For every new sample, the whole setup was cleaned with deionized water, and the felts and bipolar plates were renewed.

### Short‐stack VRFB‐system experiments

**Figure 5:**
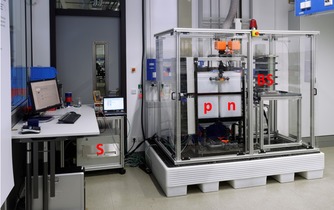
Representation of the used VRFB short‐stack system; (BS) indicates the battery stack, (n) is the negative half‐cell electrolyte tank, (p) is the positive half‐cell electrolyte tank, and (S) is the current source/sink.

In a second step, the method was applied to a short‐stack VRFB‐system, as represented in Figure 5. A stack was connected to the peripheral devices, consisting of two electrolyte tanks, two pumps (RD‐40, IWAKI Europe GmbH, Germany), a current source and sink (NL1V8C80, Höcherl & Hackl, Germany), a standard two‐compartment OCV cell, and additional devices for controlling and data logging. The stack was built of four cells with copper endplates on both sides. The flow frames were made of polypropylene with an active electrode area of 726 cm^2^. The materials for the separator, felts, bipolar plates, and OCV cell were the same as used for the lab‐scale setup. The felts were activated with oxygen plasma for 20 min. The system was flushed with nitrogen before 24 L of the commercial vanadium electrolyte [OXKEM, 1.62 m vanadium sulfate (51 % V^3+^, 49 % V^4+^) in 2 m sulfuric acid] was filled into each tank. The electrolytes were pumped through the system; afterwards, the pumps were turned off, and the system was in idle mode for 72 h to ensure that all components were in contact and soaked with the electrolytes. Subsequently, the system was repeatedly charged and discharged. Every 35 cycles the electrolytes of both half‐cells were mixed and divided into two equal portions. An electrolyte sample of the mixed electrolyte was collected from the system each time, and the AOS was determined by UV/Vis measurements (LAMBDA XLS+UV/Vis Spectrophotometer, PerkinElmer, USA) following a similar routine as described in 18. Subsequently, the potential‐step analysis was performed during the initial charging of the mixed electrolytes, and the AOS was calculated.

## Conflict of interest

*The authors declare no conflict of interest*.
